# Improving Adult Vision Through Pathway‐Specific Training in Augmented Reality

**DOI:** 10.1002/advs.202415877

**Published:** 2025-04-07

**Authors:** Yige Gao, Yulian Zhou, Qing He, Wen Wen, Peng Zhang

**Affiliations:** ^1^ State Key Laboratory of Brain and Cognitive Science Institute of Biophysics Chinese Academy of Sciences Beijing 100101 China; ^2^ University of Chinese Academy of Sciences Beijing 100049 China; ^3^ Department of Ophthalmology & Visual Science Eye & ENT Hospital Shanghai Medical College Fudan University Shanghai 200031 China; ^4^ State Key Laboratory of Medical Neurobiology and MOE Frontiers Center for Brain Science Institutes of Brain Science Fudan University Shanghai 200032 China; ^5^ Key Laboratory of Myopia and Related Eye Diseases Ministry of Health, Fudan University Shanghai 200031 China; ^6^ Shanghai Key Laboratory of Visual Impairment and Restoration Fudan University Shanghai 200031 China; ^7^ Sino‐Danish Center for Education and Research Beijing 100190 China

**Keywords:** adaptation, amblyopia, augmented reality, magnocellular and parvocellular pathways, visual disorders, visual training

## Abstract

Neurodevelopmental and neurodegenerative disorders are often associated with selective deficits in the parallel pathways of the human visual system. Effective intervention of the visual deficits is crucial for improving treatment outcomes and prognosis. In this study, a novel altered reality (AR) method is developed to improve pathway‐specific functions in human adults with normal vision and amblyopia, a common developmental vision disorder. To selectively enhance the parvocellular pathway, which is notably impaired in amblyopia, the low spatial frequency (SF) components of visual input are phase‐scrambled into fast‐flickering noise, while the high SF details remained intact. In normal participants, short‐term adaptation to this altered naturalistic environment led to selective enhancement of high SF sensitivity and visual acuity. Long‐term adaptation to a dichoptically modified environment produced long‐lasting improvement of high SF sensitivity and dominance of the weaker eye, with enhanced neural responses to high SF naturalistic stimuli. After one week of training with wearable AR glasses at home, amblyopic adults showed high treatment compliance and significant gains in visual acuity and dominance of the amblyopic eye, as well as in stereopsis. This AR‐based, pathway‐specific training method can be effective for improving visual functions in both healthy adults and patients with visual disorders.

## Graphical abstract

Traditional perceptual training approaches are limited by stimulus specificity, treatment efficacy, and patient compliance. A novel altered reality (AR) method is developed to enhance pathway‐specific functions in human adults while performing everyday activities. Dichoptic training of the AR‐based method produced robust, long‐lasting improvements of visual acuity, stereopsis, and parvocellular functions in both healthy adults and amblyopic patients.

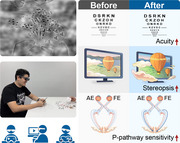

## 1 Introduction

The human visual system is organized into parallel information processing streams.^[^ ^1^, ^2^ ^]^ The two primary ones, the magnocellular (M) and parvocellular (P) pathways, are specialized to process different spatiotemporal frequencies, motion, and form ref. [3]. Specifically, parvocellular neurons have small cell bodies, thin axons, and slow conduction speed, responsible for processing fine spatial details, form, and color. In contrast, the magnocellular neurons have larger cell bodies, thicker axons and faster signal transmission, specialized in detecting large scale information and motion.

Selective deficits of the parallel pathways are well documented in several neurodevelopmental and neurodegenerative disorders. For instance, magnocellular deficits have been found in developmental dyslexia^[^ ^4^ ^]^ and glaucoma.^[^ ^5^, ^6^ ^]^ Amblyopia, also known as lazy eye, is a common developmental disorder caused by abnormal visual experiences in early childhood. The neural deficits of amblyopia are primarily parvocellular,^[^ ^7^, ^8^ ^]^ characterized by visual acuity loss and reduced contrast sensitivity to high spatial frequency (SF).^[^ ^9^ ^]^ A recent human fMRI study showed selectively reduced activity in the parvocellular layers of the lateral geniculate nucleus (LGN) of the visual thalamus.^[^ ^10^ ^]^ There has been evidence showing that neuroplasticity still exists in the adult brain.^[^ ^11^, ^12^, ^13^ ^]^ Thus, an effective training method that selectively enhances the pathway‐specific functions will not only improve the visual abilities in healthy individuals, but also aid in the treatment and prognosis of patients with pathway‐specific visual disorders.

The traditional treatment of amblyopia is occluding the fellow eye (FE) to promote functions of the amblyopic eye (AE). However, for adolescents and adults who have passed the critical period of visual development, occlusion often yields unsatisfactory results, highlighting the need for alternative therapies.^[^ ^14^, ^15^, ^16^ ^]^ Recent studies suggest that dichoptic or binocular therapies could be promising for mitigating abnormalities in binocular functions.^[^ ^17^, ^18^ ^]^ However, a recent randomized clinical trial showed that video game playing using binocular contrast‐balanced stimuli did not yield significant improvement compared to placebo game playing.^[^ ^19^ ^]^ Therefore, there still lacks an effective treatment approach targeting the monocular sensitivity and binocular interactions in the parvocellular pathway, which is particularly impaired in amblyopia.

The M and P pathways are not independent. Short‐term adaptation in one spatiotemporal frequency channel can increase the sensitivity of the other,^[^ ^20^, ^21^, ^22^ ^]^ suggesting mutual inhibitory interactions between the M and P channels.^[^ ^23^ ^]^ Furthermore, top‐down influences also play an important role in perceptual training.^[^ ^24^, ^25^ ^]^ Compared to traditional adaptation or perceptual training approaches, altered reality (AR) is a powerful method that allows more flexible controls over naturalistic statistics. Participants can have prolonged adaptation in naturalistic environments during their daily activities,^[^ ^26^, ^27^ ^]^ potentially leading to better compliance, more substantial and long‐lasting improvements in visual abilities related to their daily life.^[^ ^28^ ^]^

In the current study, we developed a novel altered reality (AR) method designed to selectively enhance visual functions of the parvocellular channel in adults with normal vision and those with amblyopia. In Experiment 1 (Exp. 1), we examined the effect of short‐term adaptation (30 minutes (min) or 4 hours (h)) in an altered environment in normal participants (N = 22). Low SF information was phase‐scrambled and presented as fast flickering noise, while the high SF components remained intact. The low SF flickers was intended to adapt the M channel, thereby enhancing the sensitivity of the P channel through disinhibition. Additionally, top‐down influence from high‐order brain regions was expected to further improve the processing of naturalistic information in the P channel while suppressing noise in the M channel.

Experiment 2 (Exp. 2) investigated whether long‐term (five days, 2 h per day) adaptation to dichoptically modified visual input could improve the weaker eye of binocularly imbalanced, but otherwise normal participants (N = 15). During adaptation, the weaker or non‐dominant eye (NDE) was exposed to the same stimuli as in Exp. 1, while the stronger or dominant eye (DE) was presented with high SF images at low signal‐to‐noise ratio (SNR). The less informative input from the dominant eye would receive reduced processing in intermediate‐ and high‐level visual areas, thereby reducing its weight in binocular interactions in the parvocellular channel through feedback modulation. Electroencephalogram (EEG) recordings were used to assess changes in pathway‐selective neural activity in the early visual cortex before and after training. In Experiment 3 (Exp. 3), we developed light‐weight wearable AR glasses, allowing adult amblyopes (N = 26) to perform AR‐based dichoptic training at home for 2 h per day over one week. Clinical and psychophysical assessments were used to measure improvements in visual abilities.

## 2 Results

### 2.1 Short‐Term AR Training Selectively Improved High SF Sensitivity and Visual Acuity

**Figure 1:**
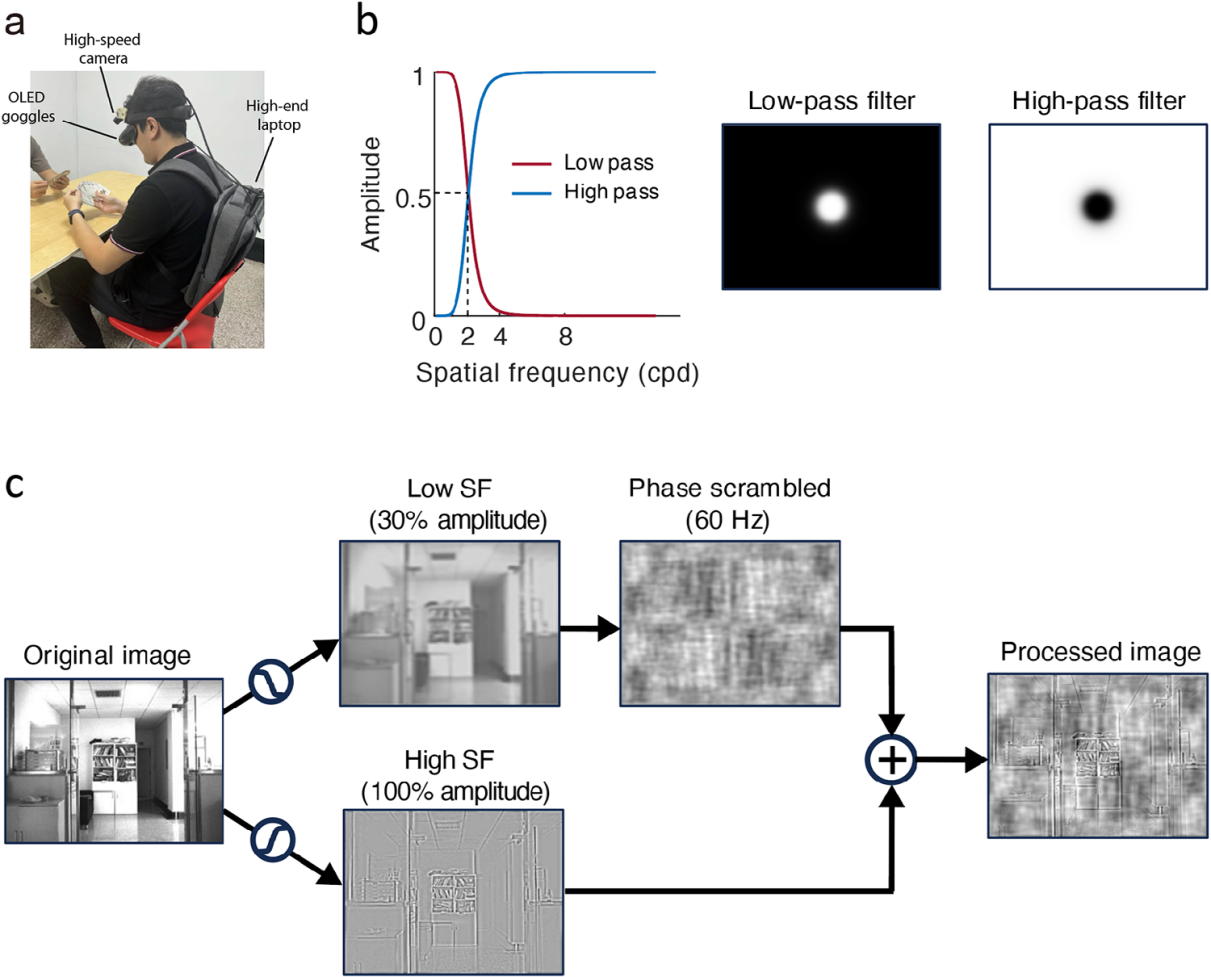
The altered reality system and image processing pipeline in Exp. 1. a) Images were captured by a high‐speed camera, processed in real time by a high‐performance laptop computer, and then presented to the participant through OLED goggles. The participant (the author Y.G.) was playing poker game with another person in this photo (Y.G. gave written consent for the publication of his images). b) The left panel shows the line plots of the Butterworth filter, with the cutoff SF at two cycles per degree (cpd). The middle and right panels show the low‐pass and high‐pass filters in the 2D Fourier domain. c) The original camera image was first low‐pass and high‐pass filtered. The amplitude of the low SF component was reduced to 30% and phase scrambled at 60 Hz, and then summed with the high SF component as the final processed image.

The AR system is composed of a high‐speed high‐definition camera, a high‐performance laptop computer with Nvidia graphics, and a pair of head‐mounted organic light‐emitting diode (OLED) goggles (**Figure** **1**a). Camera images were captured at 1440x1080 pixels at 60 Hz in grayscale, processed in real time by parallel computing algorithms (Nvidia CUDA) through high performance graphic card in the laptop, and then the processed images were presented through goggles. The image filter and processing pipeline were shown in Figure 1b,c, respectively. The cutoff frequency was selected based on the contrast sensitivity functions of the M and P channels in primates.^[^ ^2^ ^]^ Video S1 (Supporting Information) demonstrates the processed visual stimuli. During adaptation, participants can perform their daily activity, such as reading, watching movies, playing games, etc.

**Figure 2:**
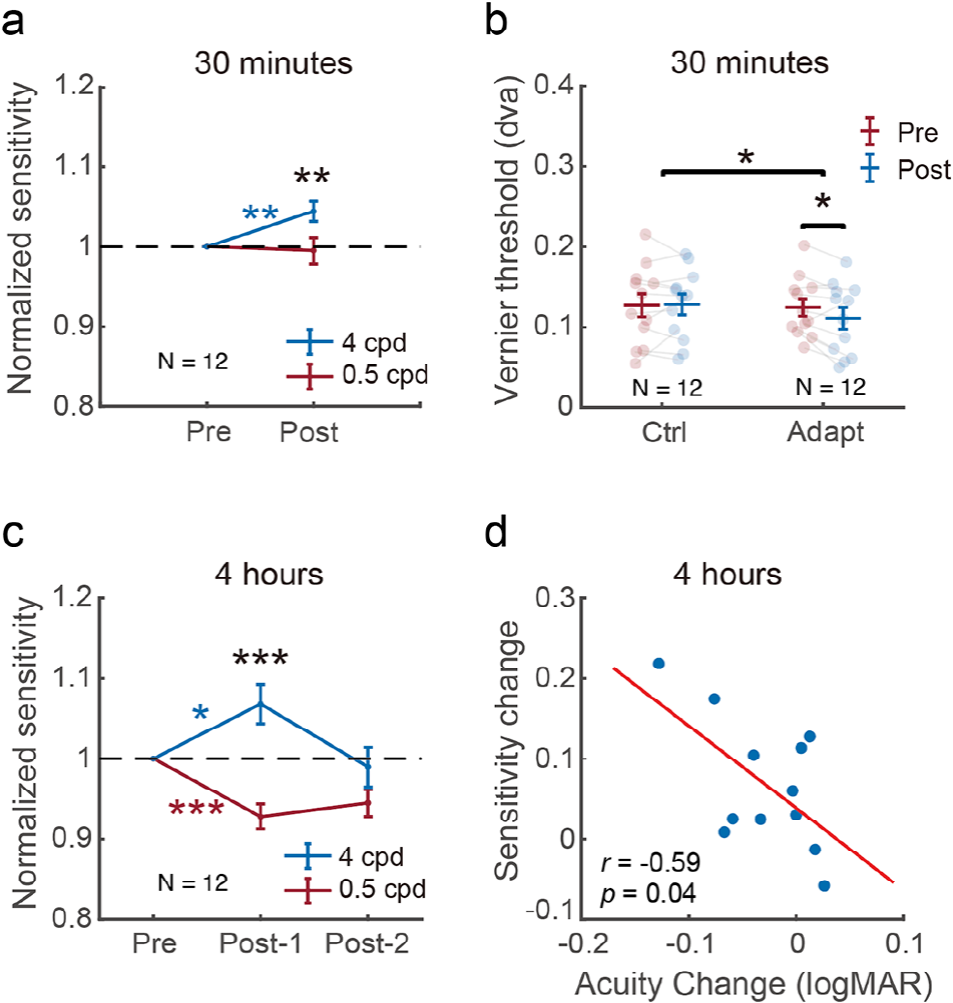
Results of Exp. 1. a) Normalized contrast sensitivity in Exp. 1a (N=12). Contrast sensitivity was defined as the reciprocal of detection threshold, and divided by that in the pre‐test session. b) Vernier acuity results in Exp 1b (N=12 in each group). dva: degrees of visual angle. c) Normalized contrast sensitivity in Exp. 1c (N=12). d) The correlation between the changes in high‐SF sensitivity and visual acuity in Exp. 1c. Each dot represents data from one participant. *, ** and *** denote *p* < 0.05, 0.01 *and* 0.001, respectively. Error bars represent ± standard error of the mean (SEM) across participants.

In Exp. 1a (N = 12), 30 min adaptation in the altered reality significantly improved the contrast sensitivity to high (4 cpd, 4.4% improvement, *t*(11) = 3.952, *p* = 0.004, *Cohen*′*s* *d* = 1.037), but not low (0.5 cpd, *t*(11) = −0.315, *p* = 0.758, Cohen's *d* = −0.091) SF gratings (**Figure** **2**a). Normalized sensitivity in the post‐test (divided by those in the pre‐test) was significantly improved in high compared to low SF condition (*t*(11) = −2.739, *p* = 0.019, *Cohen*′*s* *d* = −0.791). In Exp. 1b, Vernier acuity was measured in two group of participants in two sessions separated by 30 min either with or without adaptation in altered environment (N = 12 in each group). The results showed a significant group by session interaction ($F\left(1,11\right)=5.598,p=0.037,\eta_{p}^{2}=0.337$) (Figure 2b). The Vernier threshold was significantly reduced in the adaptation group (*t*(11) = 2.565, *p* = 0.026, *Cohen*′*s* *d* = 0.740), but not in the control group (*t*(11) = −0.209, *p* = 0.839, *Cohen*′*s* *d* = −0.060).

In Exp. 1c (N = 12), to further investigate the effect of adaptation over a longer time scale, the adaptation period was extended to four hours. Contrast sensitivity and Landolt C acuity were measured before (pre) and after (post) adaptation. Immediately after adaptation (post‐1), contrast sensitivity increased significantly by 6.8% for high SF (t(11) = 2.907, p = 0.014, Cohen's d = 0.839), whereas decreased by 7.2% for low SF (*t*(11) = −4.819, *p* < 0.001, *Cohen*′*s* *d* = −1.391) (Figure 2c). Normalized sensitivity showed highly significant difference between the two SF conditions (*t*(11) = −6.482, *p* < 0.001, *Cohen*′*s* *d* = −2.489). At the second post‐test session (post‐2, about 20 min after adaptation), contrast sensitivity at low SF was still 5.5% below the pre‐test level (*t*(11) = −3.466, *p* = 0.005, *Cohen*′*s* *d* = −1.001), while the high SF sensitivity returned to baseline (*t*(11) = −0.445, *p* = 0.665). Compared to the decline in low SF sensitivity, the improvement in high SF sensitivity was less significant and disappeared more quickly, which may be attributed to fatigue following 4 h of adaptation.^[^ ^27^ ^]^ Visual acuity measured in‐between the two post‐test sessions showed an insignificant trend of improvement (*t*(11) = 2.126, *p* = 0.057, *Cohen*′*s* *d* = 0.614). However, there was a significant correlation between the changes in high SF sensitivity (post‐test1) and visual acuity (*r* = −0.591, *p* = 0.043, Figure 2d).

These findings showed that short‐term adaptation to the altered environment selectively enhanced high SF sensitivity in the parvocellular channel and improved visual acuity.

### 2.2 Long‐Term AR‐Based Dichoptic Training Selectively Improved High SF Sensitivity and Dominance of the Weaker Eye

**Figure 3:**
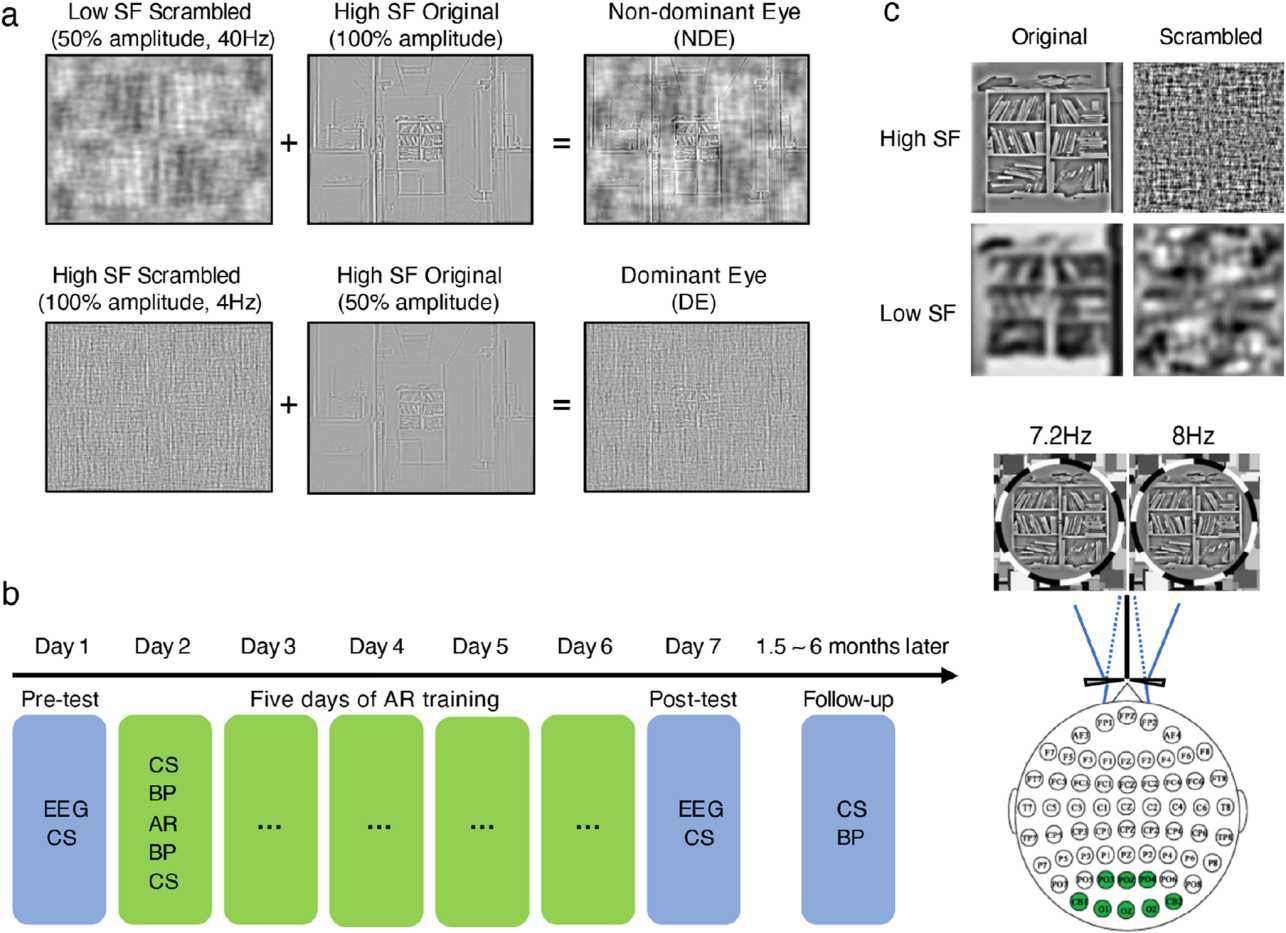
Stimuli and procedure in Exp. 2 (N=15). a) Camera image processing. b) Experimental procedure. CSF and SSVEPs were measured on day one and day seven, with five days of AR training in between. A follow‐up test was performed after 1.5 to 6 months. EEG: EEG recordings of SSVEPs, CS: contrast sensitivity measures, BP: binocular phase combination, AR: altered reality adaptation. c) Visual stimuli in the SSVEP test sessions. Original and phase scrambled images were presented at high or low SFs, either to the NDE, DE, or both eyes. A pair of prisms and a cardboard divider were used for dichoptic presentation. Green electrodes in the occipital lobe were used for data analysis.

In Exp. 2, we investigated whether long‐term dichoptic training in altered environment could selectively improve the weaker eye's sensitivity and dominance in binocularly imbalanced participants. Camera image processing is shown in **Figure** **3**a. Like in Exp. 1, the weaker or non‐dominant eye (NDE) was presented with phase‐scrambled low‐SF noise flickering at 40Hz (Low SF scrambled in Figure 3a), while high SF information (High SF original) was preserved. The dominant eye (DE) was presented with high‐SF, low SNR images. In which, phase‐scrambled high‐SF noise flickers of 100% amplitude at 4Hz (High SF scrambled) were added to the original high SF image with 50% amplitude (High SF original).

Binocularly imbalanced participants were screened from college students using the binocular phase combination task.^[^ ^29^ ^]^ A total of 15 out of 63 participants with more than 20% interocular contrast difference at the binocularly balanced point were recruited. Participants received five days of training, 2 h per day (Figure 2b). Contrast sensitivity functions (CSF) and steady‐state visually evoked potentials (SSVEPs, Figure 2c) were measured before and after five days of training. Binocular phase combination and high SF contrast sensitivity were measured before and after training in each day. To investigate whether the training effect can be maintained over a long period of time, a follow‐up test of CSF and binocular phase combination was scheduled 1.5 to 6 months after training (inter‐subject variability in the follow‐up test time was due to the COVID‐19 pandemic).

**Figure 4:**
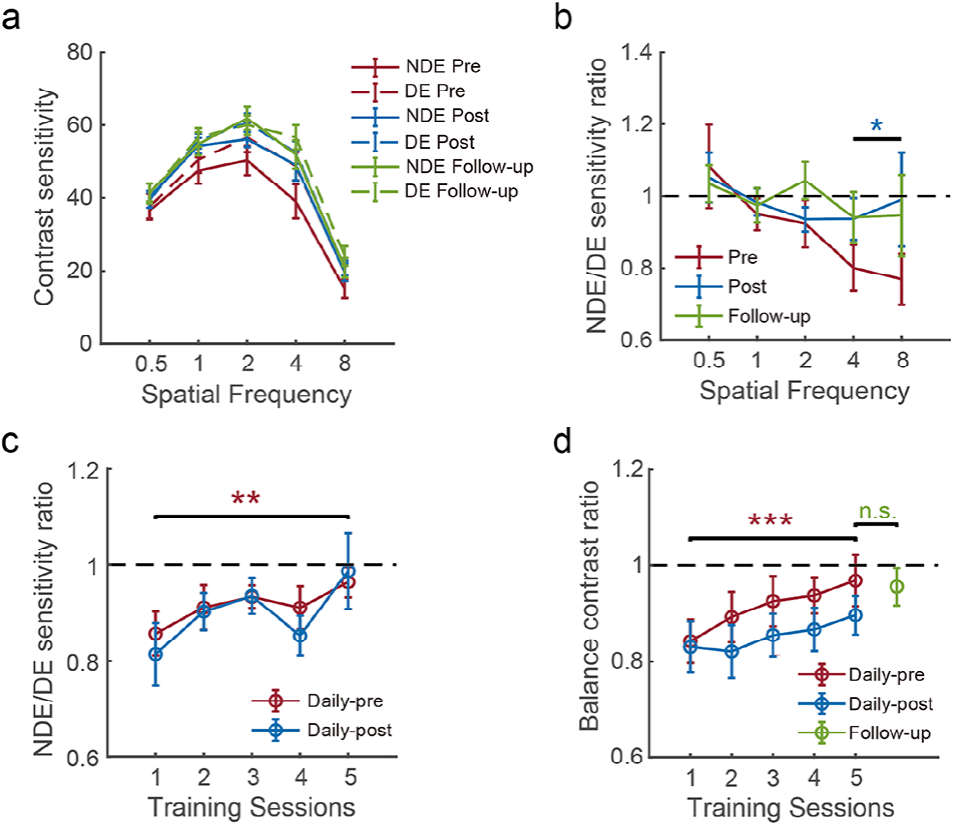
Psychophysical results of Exp. 2 (N=15). a) Contrast sensitivity functions. b) Contrast sensitivity ratio between NDE and DE. The blue asterisk indicates significant sensitivity increase at high SFs (4 and 8 cpd) in the post‐test session. c) NDE/DE contrast sensitivity ratio at high SF (4 cpd) over the training days. d) Interocularly balanced contrast ratio in binocular phase combination. *, ** and *** denote *p* < 0.05, 0.01 *and* 0.001, respectively. Error bars represent ±SEM across participants.

In **Figure** **4**a, the area under the curve (AUC) of CSF showed a significant interaction between eye (NDE/DE) and session (pre/post) ($F\left(1,14\right)=6.271,p=0.025,\eta_{p}^{2}=0.309$). A significant improvement was found for the NDE (*t* = −4.202, *p* < 0.001, *Cohen*′*s* *d* = −0.534), but not for the DE (*t* = −1.931, *p* = 0.074). When comparing the pre‐test and follow‐up sessions, the interaction between eye and session was also significant ($F\left(1,14\right)=4.596,p=0.050,\eta_{p}^{2}=0.247$), suggesting more significant improvement in NDE (*t* = −6.795, *p* < 0.001, *Cohen*′*s* *d* = −0.696) compared to DE (*t* = −2.454, *p* = 0.028, *Cohen*′*s* *d* = −0.419). These findings demonstrate a larger improvement in contrast sensitivity for the NDE compared to the DE after long‐term training in dichoptically modified naturalistic environment, and the training effect can last for months.

To better investigate the eye‐specific training effect, the contrast sensitivity ratio between NDE and DE was plotted in Figure 4b. Compared to the pre‐test, the NDE/DE sensitivity ratio in the post‐test showed a significant improvement in the high (mean sensitivity at 4 and 8 cpd, *t*(14) = 2.654, *p* = 0.019, *Cohen*′*s* *d* = 0.685), but not in the low (mean sensitivity at 0.5 and 1 cpd, *t*(14) = −0.019, *p* = 0.985) SF conditions. In the follow‐up test, there was a marginally significant effect in the high (*t*(14) = 2.022, *p* = 0.063, *Cohen*′*s* *d* = 0.522), but not in the low (*t*(14) = 0.137, *p* = 0.893) SF conditions. These results show that the eye‐specific improvement in contrast sensitivity was significant only in the high SF channel.

Figure 4c,d show the changes in high SF (4 cpd) sensitivity and ocular dominance in binocular combination over the training days. Contrast sensitivity to the high SF grating shows an increasing improvement over days ($F\left(4,56\right)=2.917,p=0.029,\eta_{p}^{2}=0.172$), with a highly significant improvement (12.5%) in the last compared to the first day of training (daily pre‐test sensitivity difference: *t*(14) = −3.551, *p* = 0.003, *Cohen*′*s* *d* = −0.917). The NDE dominance in binocular phase combination also improved significantly over the training days ($F\left(4,56\right)=3.656,p=0.010,\eta_{p}^{2}=0.207$), leading to a large improvement in the last compared to the first day of training (daily pre‐test sensitivity difference: *t*(14) = −4.039, *p* = 0.001, *Cohen*′*s* *d* = −1.043). The improvement in NDE dominance was maintained in the follow‐up test (compared to the first day of training: *t*(14) = −2.570, *p* = 0.022, *Cohen*′*s* *d* = −0.663; compared to the last day of training: *t*(14) = 0.213, *p* = 0.853). Interestingly, NDE dominance decreased immediately after training within each day ($F\left(1,14\right)=6.417,p=0.024,\eta_{p}^{2}=0.314$). This is likely a short‐term effect of homeostatic plasticity, as suggested by previous studies on monocular form deprivation.^[^ ^30^, ^31^, ^32^ ^]^ This strong aftereffect in the opposite direction may have counteracted the sensitivity improvement as in Exp. 1, leading to no significant change in sensitivity balance between the two eyes after training (Figure 4c). These behavioral results demonstrate a long‐lasting improvement in high‐SF sensitivity and dominance of the weaker eye following five‐days of adaptation to dichoptically modified naturalistic environment.

**Figure 5:**
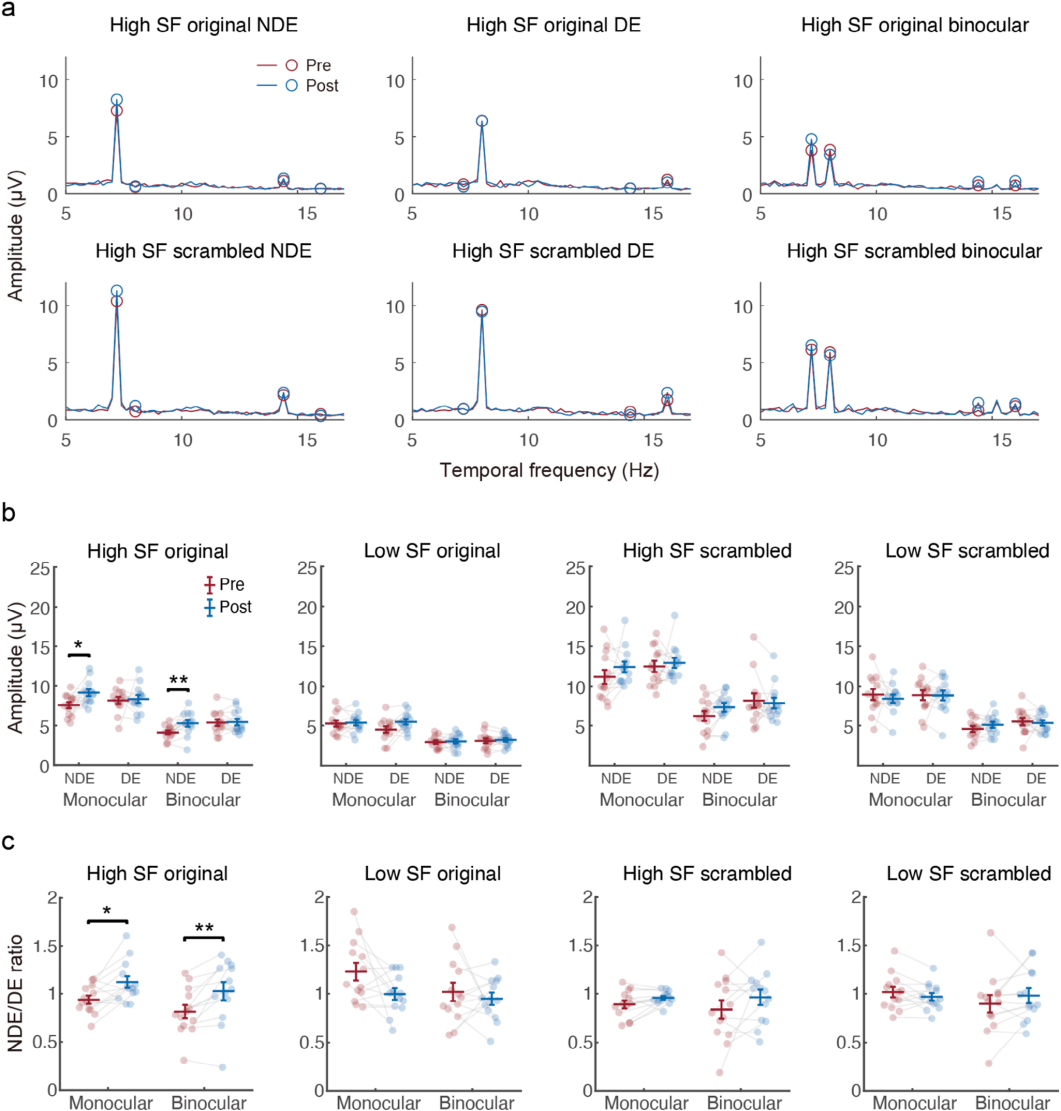
SSVEP results of Exp. 2 (N=15). a) Group‐averaged amplitude spectra to high SF stimuli with original or scrambled phases presented to NDE (7.2 Hz), DE (8 Hz) or both eyes. Figure S1 (Supporting Information) shows the amplitude spectra when NDE and DE stimuli were presented at 8 and 7.2 Hz, respectively. Figure S2 (Supporting Information) shows the results for low SF stimuli. b) Normalized SSVEP amplitude across all stimulus conditions. The SSVEP amplitude in each stimulus conditions was divided by the mean across all stimulus conditions. c) SSVEP response ratio between NDE and DE conditions. * and ** denote *p* < 0.05 *and* 0.01, respectively. Error bars represent ±SEM across participants.

In the SSVEP test sessions, we directly measured neural activity in the visual cortex to original and phase‐scrambled images at low and high SFs (Figure 3c). Processed camera images were presented to the two eyes at slightly different temporal frequencies, on and off flickering at 7.2 or 8 Hz. Amplitude spectra to the high SF stimuli presented to the NDE, DE and both eyes are shown in **Figure** **5**a (Figures S1 and S2, Supporting Information shows the amplitude spectra for other stimulus conditions). In Figure 5b, only the SSVEP amplitude to the high SF original image showed a significant eye by session interaction (*F*(1, 14) = 17.458, *p* = 0.005 Holm corrected, $\eta_{p}^{2}=0.593$; *p* > 0.05 for other stimulus conditions). Further analysis revealed a significant increase in amplitude for the NDE ($F\left(1,14\right)=8.675,p=0.012,\eta_{p}^{2}=0.420$), in both monocular (*t*(12) = −2.534, *p* = 0.026, *Cohen*′*s* *d* = −0.703) and binocular (*t*(12) = −3.196, *p* = 0.008, *Cohen*′*s* *d* = −0.886) conditions. In contrast, DE responses showed no significant difference before and after training ($F\left(1,14\right)=0.056,p=0.817,\eta_{p}^{2}=0.005$). The NDE/DE response ratio also significantly increased in both monocular (*t*(12) = −2.958, *p* = 0.012, *Cohen*′*s* *d* = −0.821) and binocular (*t*(12) = −3.778, *p* = 0.003, *Cohen*′*s* *d* = −1.048) conditions (Figure 5c). These findings provide direct neuroimaging evidence that NDE sensitivity to high SF naturalistic input was significantly enhanced in the early visual cortex.

### 2.3 One Week of AR Training at Home Improved Visual Acuity and Dominance of the Amblyopic Eye and Stereopsis in Adult Amblyopes

**Figure 6:**
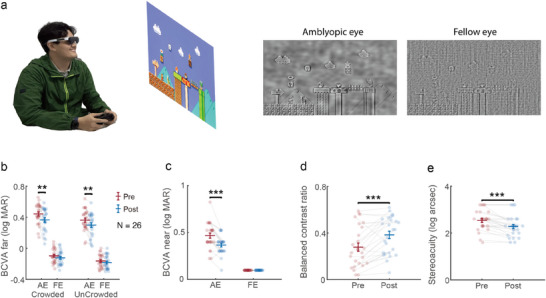
Clinical and psychophysical results of Exp. 3 (N=26). a) In this demo, the participant was playing video game while wearing AR glasses. Right panels show the processed images presented to the amblyopic eye and the fellow eye. b) Best‐corrected visual acuity (BCVA) results with single tumbling E at a far distance (2.2 m). c) BCVA results at a near distance (30 cm). d) Binocularly balanced contrast ratio in binocular phase combination task. e) Stereoacuity measured with Titmus Fly chart. *, **, and *** denote *p* < 0.05, 0.01 *and* 0.001, respectively. Error bars represent ±SEM across participants.

In Exp. 3, we investigated whether AR adaptation in dichoptically modified environment could improve the visual acuity and dominance of the amblyopic eye in adult patients. To facilitate training at home, we developed lightweight wearable AR glasses (**Figure** **6**a, see Experimental Section for details). A total of 26 adults with monocular amblyopia received dichoptic training with the AR method at home for one week (or 7 days), 2 h per day. Demographics and baseline characteristics were summarized in Table S1 (Supporting Information). During adaptation, images presented to the amblyopic eye (AE) and fellow eye (FE) of amblyopic patients were identical as those presented to the NDE and DE of normal participants in Exp. 2 (Figure 3a). Before and after training, the best corrected visual acuities (BCVA) in crowded and uncrowded conditions, with and without a square frame surrounding the target, were measured by the Tumbling E Acuity test at a far viewing distance of 2.2 m. BCVA at a near distance of 30 cm was measured with the standard logarithmic E chart. Dominance of the amblyopic eye in binocular vision was measured using the binocular phase combination paradigm. Stereoacuity was assessed using the Titmus fly test.

BCVA at a far distance (Figure 6b) showed a significant eye (AE/FE) by session (Pre/Post) interaction ($F\left(1,25\right)=9.291,p=0.005,\eta_{p}^{2}=0.002$), indicating greater improvement for the AE compared to the FE. BCVA for the AE improved significantly in both crowded (from 0.442 ± 0.129 to 0.366 ± 0.147 logMAR, *W* = 9, *z* = −3.924, *p* < 0.001, *RBC* = −0.935, Wilcoxon signed‐rank test), and uncrowded (from 0.363 ± 0.138 to 0.299 ± 0.149 logMAR, *t*(25) = −3.628, *p* = 0.001, *Cohen*′*s* *d* = −0.712) conditions. The BCVA for the FE also showed a small but significant improvement (crowded: *t*(25) = −2.857, *p* = 0.008, *Cohen*′*s* *d* = −0.560; uncrowded: *t*(25) = −2.492, *p* = 0.02, *Cohen*′*s* *d* = −0.489). In Figure 6c, BCVA at a near distance also showed a significant eye (AE/FE) by session (Pre/Post) interaction ($F\left(1,25\right)=36.927,p<0.001,\eta_{p}^{2}=0.596$), with AE acuity improved significantly (from 0.466 ± 0.027 to 0.365 ± 0.019 logMAR, *t*(25) = −6.077, *p* < 0.001, *Cohen*′*s* *d* = −1.192), while FE acuity remained at the upper limit of the visual acuity chart (‐0.097 logMAR) for all participants. In the binocular phase combination test (Figure 6d), AE dominance showed a highly significant improvement (*t*(25) = 4.480, *p* < 0.001, *Cohen*′*s* *d* = 0.879). Finally, stereoacuity showed a highly significant improvement from 2.540 ± 0.085 to 2.281 ± 0.088 log arcsec (*W* = 171, *z* = −3.724, *p* < 0.001, *RBC* = −1.000) (Figure 6e). Home‐based training with wearable AR glasses achieved extremely high treatment compliance: patients finished 102.16% ± 2.22% of the prescribed duration and achieved a session completion rate of 79.26% ± 4.32% (the percentage of sessions that met or exceeded the prescribed duration, see Figure S3 (Supporting Information) for the compliance results for all participants).

## 3 Discussion

In the current study, we developed a novel altered reality method to selectively improve functions of the parvocellular pathway in human adults with normal vision and those with amblyopia. For normal participants, short‐term adaptation in the altered environment improved both visual acuity and high SF sensitivity, while long‐term dichoptic training produced a long‐lasting improvement of the weaker eye. For amblyopic patients, one week of home‐based training with wearable AR glasses showed high treatment compliance, and robust improvements in visual acuity and dominance of the amblyopic eye, as well as in stereopsis. This pathway‐specific AR training method shows great promise for improving visual performance in both healthy individuals and patients with visual disorders.

In previous studies, short‐term spatial frequency adaptation reduced the contrast sensitivity around the adapted frequency,^[^ ^33^ ^]^ and enhanced the sensitivity of spatial frequencies further away from the adapted frequency.^[^ ^22^, ^23^ ^]^ Blur adaptation sharpens visual perception even without improvement in high SF sensitivity,^[^ ^21^, ^34^ ^]^ and adaptation to motion or flickers showed a similar effect.^[^ ^20^, ^35^ ^]^ In our study, short‐term adaptation to low SF noise flickers with intact high SF naturalistic information improved both high SF sensitivity and visual acuity (Figure 2), while reducing the contrast sensitivity for low SF. These findings demonstrate that our AR‐based method is highly effective in selectively modulating the sensitivity and function of the magnocellular and parvocellular pathways.

More importantly, long‐term dichoptic training in the altered environment produced robust and long‐lasting improvement in visual functions closely related to the P pathway for the weaker eye in both healthy (Figure 4) and amblyopic adults (Figure 6). Furthermore, EEG recordings showed that AR training in the naturalistic environment selectively enhanced neural activity to high SF naturalistic stimuli in the early visual cortex (Figure 5). Thus, in addition to the low‐level adaptation mechanisms, our AR‐based training effects should have also involved top‐down influences from high‐level brain regions for naturalistic processing, which could be a possible reason for the long‐lasting improvement. In support of this, recent studies showed that top‐down attention and feedback modulations can be eye‐specific,^[^ ^36^, ^37^ ^]^ and may influence ocular dominance plasticity.^[^ ^38^ ^]^ In addition, there has been evidence showing that top‐down modulations from high‐level brain regions play an important role in visual perceptual learning (VPL).^[^ ^25^, ^39^ ^]^ While we did not separately measure the long‐term effects of adding phase‐scrambled high SF noise to the dominant eye and low SF noise flickers to the non‐dominant eye, previous studies have shown that monocular phase deprivation does not change ocular dominance the following day.^[^ ^30^, ^32^ ^]^ Thus, the observed effects are likely attributed to the dichoptic manipulations of low and high SF channels. To further elucidate the plasticity mechanisms, these effects should be measured separately in future studies.

Finally, home‐based training with wearable AR glasses enabled strong interactions with the altered visual environment while performing everyday activities, including social interactions. Compared to traditional training methods such as monocular patching or VPL, our AR‐based methods can have better treatment efficacy and patient compliance (Figure S3, Supporting Information). In future studies, the image processing parameters, such as the cutoff spatial frequency, temporal frequency, and contrast ratio between different spatiotemporal frequency and ocular channels, can be individually optimized to further improve the training effect. In a recent randomized clinical trial study,^[^ ^19^ ^]^ home‐based binocular treatment playing a falling‐block video game for older children and adults showed no significant improvement compared to the placebo game‐play group. Patient compliance, in term of training duration, was much lower than in the current study. Although we did not directly compare with the binocular contrast‐balancing method, it is likely that our AR method targeting the parvocellular pathway may produce a stronger training effect.

In addition to amblyopia, our methods could also be useful for interventions for other neurodevelopmental and neurodegenerative visual disorders. For example, magnocellular deficits have been identified in developmental dyslexia, including contrast detection and motion discrimination impairments,^[^ ^40^ ^]^ abnormal functional activity and connections in the magnocellular pathway,^[^ ^41^, ^42^, ^43^ ^]^ and cell atrophy in the M layers of the LGN of the thalamus.^[^ ^4^ ^]^ In glaucoma, studied have shown loss and atrophy of parasol ganglion cells in the retina,^[^ ^6^ ^]^ loss and atrophy of M cells of the LGN,^[^ ^5^, ^44^ ^]^ and selective reduction of BOLD response in the M layers of the LGN in an early stage of the disease.^[^ ^45^ ^]^ Visual perceptual training has also been found to improve visual deficits in both developmental dyslexia^[^ ^46^ ^]^ and glaucoma.^[^ ^47^ ^]^ Thus, similar to the AR training approach designed to target the P pathway, adaptation to phase‐scrambled high SF noise with intact low SF information (see Video S2, Supporting Information for demonstration) with the AR method could be an effective intervention for selectively enhancing magnocellular function in patients with  disorders in the M pathway.

To summarize, we developed a novel altered reality (AR) training method that can produce robust and long‐lasting improvement in parvocellular and magnocellular pathway‐specific visual functions in human adults while performing everyday activities. This pathway‐specific AR training approach holds great promise for improving visual functions in both healthy individuals and patients with visual disorders, including but not limited to amblyopia, glaucoma, and developmental dyslexia.

## 4 Experimental Section

### Participants

A total of 27 healthy adults (24.96 (mean) ± 4.31 (SD) years of age, 15 females) participated in Exp. 1 (N = 12, 24 and 12 in Exp.1a, Exp. 1b, and Exp. 1c, respectively). In Exp. 2, another 15 interocularly imbalanced participants (23.67 ± 3.18 years of age, eight females) with at least 20% interocular contrast difference at the balanced point in the binocular phase combination test were recruited. In Exp. 1 and Exp. 2, participants needed to complete a visual acuity test before experiment and wear corrective glasses to ensure that each eye reached 0.6 decimal or higher in the test sessions. The experimental procedures were approved by the ethical review board of Institute of Biophysics, Chinese Academy of Sciences (2017‐IRB‐003). Written informed consent was obtained from all participants prior to their participation in the study.

In Exp. 3, 26 adults diagnosed with monocular amblyopia were included (26.92 ± 5.17 years of age, 18 females). Inclusion criteria were 18–45 years of age, BCVA of the AE in the range of 0.1–1.0 logMAR and no more than 0.1 logMAR for the FE, and at least three months of optical correction before the AR training. Exclusion criteria were eccentric fixation, nystagmus, manifest strabismus, history of ocular trauma, optic nerve disease, corneal opacity, cataract, glaucoma, and other organic eye diseases affecting visual acuity, neurological, psychiatric diseases, and other serious systemic diseases. The informed consent form was developed in accordance with the Declaration of Helsinki for Medical Research Involving Human Participants (the 75th WMA General Assembly, 2024). Written informed consent form was signed by every participant after they had fully understood this study. This study was approved by the ethical committee of Eye & ENT Hospital of Fudan University (Ethical code: [2023] No. 2023020).

### Altered Reality System

In Exp. 1 and Exp. 2, grayscale images were acquired in Y800 color format at 1440×1080 resolution and 60 Hz frame rate using a high‐definition camera (DMK 37AUX273, Imaging Source) with a 49‐degree field‐of‐view lens (BT‐118C0620MP, MicroVision). Camera images were processed in real‐time by parallel computing toolbox (supported by Nvidia CUDA) in Matlab using a high‐performance laptop computer with Nvidia GTX 1060 graphics card. Images were fast Fourier transformed into real and imagery parts. The cutoff frequency (50% attenuation) of the 2‐D Butterworth filter was 2 cpd. The phase of low‐SF component was replaced by the phase of white noise images with a uniform distribution from ‐pi to pi. The real part of the inverse fast Fourier transformed image was taken as the final image. The phase‐scrambling method preserves the conjugate nature of data in the 2D Fourier domain, which preserved the energy in the real component of image following inverse FFT.^[^ ^27^, ^30^ ^]^ While the phase scrambling method might not precisely match the neural activity in an early visual area,^[^ ^48^ ^]^ it effectively eliminates all meaningful information in the image, making it suitable for the purposes as a noise image in the current study. Processed images were presented to OLED goggles (GOOVIS G2pro), with 53‐degree field of view, 1920x1080 pixel resolution in each eye and 60 (Exp. 1) or 40 Hz (Exp. 2) frame rate. Image presentations were programmed using Psychtoolbox (version 3.0) in Matlab.^[^ ^49^ ^]^ To ensure the accuracy and reliability of the AR method, validation tests were performed before the real experiments. To validate the accuracy of spatial filtering, gratings were printed out on a piece of paper and positioned at a distance corresponding to spatial frequencies of 0.5, 2, and 4 cpd on the goggles' display. The filtered images were then visually inspected while wearing the goggles to confirm the accuracy of spatial filtering. Additionally, the framerate was calculated and displayed on the goggles display in real time to confirm the stability of filtering process.

In Exp. 3, the custom‐built AR glasses consist of two 0.71‐inch AMOLED micro screen, with 1920×1080 resolution at 60Hz refresh rate for each eye, and a 52‐degree field of view. The CMOS camera (GC02B10) integrated to the AR system acquired full HD (1920×1080 resolution) images in MJPG format at a maximum frame rate of 60 Hz with a 47‐degree field of view. Image processing and presentations were programmed using OpenGL with Nvidia CUDA SDK, computed in real time on a high‐performance laptop computer with Nvidia GeForce GTX 1660 graphics card. Image processing pipelines in the AE was identical as in Exp. 2, while parameter settings for the FE were individualized according to the baseline measures in binocular phase combination. In which, the amplitude of phase‐scrambled high‐SF noise flickers was 1/3 of balanced contrast ratio, while the amplitude for original high SF components was 1/6 of balanced contrast ratio. Due to limited processing bandwidth, the final framerate for presentation was 40 Hz.

### Behavioral Measures

Stimuli were generated using Psychtoolbox in Matlab. In Exp.1a, stimuli were presented with gamma‐corrected LCD monitor (Display++ of Cambridge system) in 1920*1080 resolution and 120 Hz refresh rate, at a viewing distance of 2 m. In Exp. 1b, Exp. 1c, and Exp. 2, stimuli were presented on a 21‐inch Trinitron CRT monitor (NESOJXC FP210A) calibrated by Bit# stimulus processor (Cambridge system), with 2048*1536 resolution and 60 Hz refresh rate. The viewing distance was 1.3 m.

In Exp. 1a, contrast sensitivities to high (4 cpd) and low SF (0.5 cpd) sinewave gratings were measured before and after training using the adjustment method. After pressing a button, a sinewave grating (4 degrees in diameter) was briefly presented with a pure tone for 200 ms either to the left or to the right side of fixation point at 7.5 degrees eccentricity. Participants pressed a button to increase the contrast until they could detect the grating, and then reported its location. A total of 25 trials were collected for each SF in each session.

In Exp. 1b, Vernier acuity was measured at the same eccentricity as in Exp. 1a, either in the left or the right visual field balanced across participants. Vernier threshold at 82% accuracy was measured with a two alternatives forced‐choice (2‐AFC) 3‐down‐1‐up staircase procedure. In each trial, two vertical bars with slight horizontal misalignment were presented for 200 ms at the test location, participants reported whether the top bar shifted to the left or to the right side of the bottom bar. There were 50 trials in each staircase, and a total of four staircases were measured in each session.

In Exp. 1c, contrast sensitivity was measured in the central visual field with a 6‐degree sinewave grating tilted 45 or −45 degrees from vertical using the method of adjustment. Subjects increased the contrast of the grating until they can report its orientation. A total of 25 trials were measured for each SF in each session. Visual acuity was assessed by Landolt C acuity test in FrACT.^[^ ^50^ ^]^ A total of 96 trials were measured in the pre‐ and post‐test sessions. In the post‐test sessions, visual acuity was measured in‐between the post‐1 and post‐2 sessions.

In Exp. 2, contrast sensitivity functions were measured at five SFs (0.5, 1, 2, 4, and 8 cpd) in NDE and DE in the pre‐ and post‐test sessions (the 1st and the 7th day), using a 2‐AFC 3‐down‐1‐up staircase procedure. In each trial, a sinewave grating (6 degrees in diameter) was presented to the left or right side of fixation at 3 degrees eccentricity for 200 ms. Participants need to report the location of the grating by pressing one of two buttons. Trials from different conditions (2 eyes × 5 SFs) were intermixed in a pseudorandom order. A total of 100 trials (two staircases) were measured for each condition. Binocular phase combination and the contrast sensitivity to high SF (4 cpd) grating were measured before and after the 2‐h adaptation in each training day (from the 2nd to the 6th day). In the binocular phase combination test,^[^ ^29^ ^]^ two horizontal sinewave gratings (0.46 cpd) were dichoptically presented one to each eye, with 45 degrees phase difference (shifted 22.5 degrees in upward or downward direction). The NDE contrast was fixed at 64%, while the DE contrast varied from 0 to 100%. Participants need to adjust a black line to the middle of the dark band in the binocularly combined grating. 12 trials were collected in each session. Interocular contrast ratio at the balanced point with zero phase shift was derived from the fitted psychometric curve. The method of adjustment was used to measure the contrast sensitivity at 4 cpd. A total of 24 trials were collected for each eye in each session.

In Exp. 3, the best‐corrected visual acuity (BCVA) at a near viewing distance (30 cm) was measured with standard logarithmic E chart, and the BCVA at a far distance (2.2 m) was measured with single tumbling E target with or without two‐gap surrounding square. CSFs and binocular phase combination were measured using the same stimuli and procedure as described in Exp. 2. Stereoacuity was measured with Titmus Fly chart at a viewing distance of 30 cm, and results in arcsec were transformed to log units for analysis.^[^ ^51^ ^]^

### EEG Recordings

### Visual Stimuli

Visual stimuli were presented on a high refresh rate and high‐resolution LCD monitor (Asus Swift PG278QR), at 2560*1440 pixel resolution and 144‐Hz refresh rate. The distance between the monitor and the participant was 85 cm. In the pre‐ and post‐test sessions in Exp. 2 (the 1st and the 7th day), SSVEPs were recorded to naturalistic stimuli presented in different SF (high/low), phase (original/scrambled), and ocular (NDE/DE/binocular) conditions, either at 7.2 or 8 Hz. Images were normalized to the same luminance and root‐mean‐squares contrast. Flicker stimuli were on and off presented at 7.2 and 8 Hz, presented one to each eye during monocular and binocular stimulus presentations. The tagging frequencies with specific eyes was balanced: 7.2 Hz for the left eye and 8 Hz for the right eye, and vice vera. Therefore, the EEG stimulation included a total of 2 SFs (high/low) × 2 phase conditions (original/scrambled) × 3 ocular conditions (NDE/DE/binocular) × 2 temporal frequencies (7.2/8 Hz) = 24 stimulus conditions. Stimuli were dichoptically presented with a pair of prism glasses and a cardboard divider.^[^ ^52^, ^53^ ^]^ A pair of circular frames with mosaic patterns from outside were used to assist fusion between the two eyes. Subject pressed a button to start a trial. They were instructed to keep fixation and to reduce blinks and eye movements during the 6‐s stimulus presentation period. 24 stimulation conditions in a pseudo randomized order constituted a block, each test session contained six blocks.

### Data Acquisition

EEG data were acquired using a 64‐channel NeuroScan system (SynAmps RT amplifier, 64‐channels Quik‐Cap with 10‐20 electrode placement), recorded in AC mode with a high‐pass filter from 0.05 Hz and digitized at 1000 Hz. The ground electrode positioned at AFz, and the reference electrode positioned between Cz and CPz. Data from all 64 channels were recorded. The resistance values of all channels were maintained below 8*k*ω throughout the experiment.

### Data Analysis

EEG data were processed using the EEGLAB toolbox in Matlab.^[^ ^54^ ^]^ Data were bandpass filtered from 1 to 40 Hz, and epoched into 6‐s trials. Data from the first second of each trial were discarded to avoid the transient response following stimulus onset. Trials with dropped frames were removed. Oz and the six channels centered around Oz (PO3, POz, PO4, O1, O2, CB1, and CB2) were used for further analysis. Trials in the same condition were averaged in time domain before the fast Fourier transform (FFT) for spectrum analysis. The amplitudes of SSVEPs to the 7.2 and 8 Hz stimuli were averaged for each condition. The SSVEP amplitudes for the fundamental and the second‐order harmonic were summed together for statistical analysis.

### Statistical Analysis

The distributions of continuous variables were presented as mean ± SD. Repeated measures ANOVA were used to assess the statistical significance of the main effects, e.g., session (Pre/Post), SF (low/high), eye (NDE/DE, or AE/FE), and their interactions. The multiple comparison corrections for the following paired t‐tests used the Holm‐Bonferroni method. Based on the rationale of the seminal paper by Levin and colleagues,^[^ ^55^ ^]^ post‐hoc t‐tests followed by a significant two‐way interaction did not need further correction for family‐wise errors. Partial eta squares ($\eta_{p}^{2}$) and *Cohen*′*s* *d* were reported the effect size for the F‐test in ANOVA and t‐test, respectively. Wilcoxon signed‐rank test for paired data was used if the data significantly deviated from a normal distribution, and rank‐biserial correlation (RBC) was reported as the effect size. P values <0.05 with two‐sided testing were considered statistically significant. Statistical tests in this study were performed using JASP (v0.14.1). The sample sizes (*n* ⩾ 12 in all experiments) provide at least 80% power to detect a strong effect (*Cohen*′*s* *d* > 1) as suggested by previous studies of dichoptic training in amblyopia using similar psychophysical tasks.

## Conflict of Interest

The authors declare no conflict of interest.

## Supporting information

Supporting Information

Supplemental Video 1

Supplemental Video 2

## Data Availability

The data that support the findings of this study are available on request from the corresponding author. The data are not publicly available due to privacy or ethical restrictions.

## References

[1] P. Lennie , Vis. Res. 1980, 20, 561. 7434593 10.1016/0042-6989(80)90115-7

[2] W. H. Merigan , J. H. Maunsell , Annu. Rev. Neurosci. 1993, 16, 369. 8460898 10.1146/annurev.ne.16.030193.002101

[3] M. Livingstone , D. Hubel , Science (New York, N.Y.) 1988, 240, 740. 3283936 10.1126/science.3283936

[4] M. S. Livingstone , G. D. Rosen , F. W. Drislane , A. M. Galaburda , Proc. Natl. Acad. Sci. USA 1991, 88, 7943. 1896444 10.1073/pnas.88.18.7943 PMC52421

[5] N. Gupta , T. Ly , Q. Zhang , P. L. Kaufman , R. N. Weinreb , Y. H. Yücel , Exp. Eye Res. 2007, 84, 176. 17094963 10.1016/j.exer.2006.09.013

[6] J. E. Morgan , J. Glaucoma 2002, 11, 365. 12169976 10.1097/00061198-200208000-00015

[7] A. Hendrickson , J. Movshon , H. Eggers , M. Gizzi , R. Boothe , L. Kiorpes , J. Neurosci. 1987, 7, 1327. 3033169 10.1523/JNEUROSCI.07-05-01327.1987 PMC6568823

[8] R. F. Hess , B. Thompson , G. A. Gole , K. T. Mullen , J. Neurophysiol. 2010, 104, 475. 20463193 10.1152/jn.01060.2009

[9] D. M. Levi , Vis. Res. 2020, 176, 118. 32866759 10.1016/j.visres.2020.07.014 PMC7487000

[10] W. Wen , Y. Wang , J. Zhou , S. He , X. Sun , H. Liu , C. Zhao , P. Zhang , Cell Rep. 2021, 37, 110117. 34910903 10.1016/j.celrep.2021.110117

[11] B. Dosher , Z.‐L. Lu , Annu. Rev. Vision Sci. 2017, 3, 343. 10.1146/annurev-vision-102016-061249 PMC6691499 28723311

[12] C. D. Gilbert , W. Li , V. Piech , J. Physiol. 2009, 587, 2743. 19525560 10.1113/jphysiol.2009.171488 PMC2718234

[13] W. Li , Annu. Rev. Vision Sci. 2016, 2, 109. 10.1146/annurev-vision-111815-114351 28532348

[14] F. Kishimoto , C. Fujii , Y. Shira , K. Hasebe , I. Hamasaki , H. Ohtsuki , Jpn. J. Ophthalmol. 2014, 58, 26. 24158452 10.1007/s10384-013-0279-z

[15] W. H. Ridder Iii , R. Patel , Y.‐X. Li , U. Staubli , Clin. Ophthalmol. 2023, 17, 1847. 37405009 10.2147/OPTH.S410800 PMC10317545

[16] M. M. Scheiman , R. W. Hertle , R. W. Beck , A. R. Edwards , E. Birch , S. A. Cotter , E. R. Crouch Jr , O. A. Cruz , B. V. Davitt , S. Donahue , Arch. Ophthalmol. 2005, 123, 437. 15824215 10.1001/archopht.123.4.437

[17] J. Li , B. Thompson , D. Deng , L. Y. Chan , M. Yu , R. F. Hess , Curr. Biol. 2013, 23, R308. 23618662 10.1016/j.cub.2013.01.059

[18] T. L. Ooi , Y. R. Su , D. M. Natale , Z. J. He , Curr. Biol. 2013, 23, R309. 23618663 10.1016/j.cub.2013.03.004 PMC6485254

[19] T. Y. Gao , C. X. Guo , R. J. Babu , J. M. Black , W. R. Bobier , A. Chakraborty , S. Dai , R. F. Hess , M. Jenkins , Y. Jiang , L. S. Kearns , L. Kowal , C. S. Y. Lam , P. C. K. Pang , V. Parag , R. Pieri , R. N. Raveendren , J. South , S. E. Staffieri , A. Wadham , N. Walker , B. Thompson , JAMA Ophthalmol. 2018, 136, 172. 29302694 10.1001/jamaophthalmol.2017.6090 PMC6584719

[20] D. H. Arnold , J. D. Williams , N. E. Phipps , M. A. Goodale , Proc. Natl. Acad. Sci. 2016, 113, 12556. 27791115 10.1073/pnas.1609330113 PMC5098642

[21] M. Mon‐Williams , J. R. Tresilian , N. C. Strang , P. Kochhar , J. P. Wann , Proc. R. Soc. London. Series B: Biol. Sci. 1998, 265, 71. 10.1098/rspb.1998.0266 PMC1688761 9470217

[22] N. Rajeev , A. Metha , Invest. Ophthalmol. Vis. Sci. 2010, 51, 1242. 19797213 10.1167/iovs.09-3965

[23] K. K. De Valois , Vis. Res. 1977, 17, 1057. 595415 10.1016/0042-6989(77)90010-4

[24] Z. Başgöze , A. P. Mackey , E. A. Cooper , Curr. Biol. 2018, 28, R1406. 30562537 10.1016/j.cub.2018.10.024

[25] T. Watanabe , Y. Sasaki , Annu. Rev. Psychol. 2015, 66, 197. 25251494 10.1146/annurev-psych-010814-015214 PMC4286445

[26] M. Bao , S. A. Engel , Curr. Dir. Psychol. Sci. 2019, 28, 574.

[27] P. Zhang , M. Bao , M. Kwon , S. He , S. A. Engel , Curr. Biol. 2009, 19, 1956. 19896377 10.1016/j.cub.2009.10.018

[28] M. Bao , B. Dong , L. Liu , S. A. Engel , Y. Jiang , Psychol. Sci. 2018, 29, 14. 29160741 10.1177/0956797617728126

[29] J. Ding , G. Sperling , Proc. Natl. Acad. Sci. 2006, 103, 1141. 16410354 10.1073/pnas.0509629103 PMC1347993

[30] J. Bai , X. Dong , S. He , M. Bao , Neuroscience 2017, 352, 122. 28391010 10.1016/j.neuroscience.2017.03.053

[31] H.‐W. Kim , C.‐Y. Kim , R. Blake , Curr. Biol. 2017, 27, 884. 28262490 10.1016/j.cub.2017.01.063

[32] J. Zhou , A. Reynaud , R. F. Hess , Proc. R. Soc. B: Biol. Sci. 2014, 281, 20141717. 10.1098/rspb.2014.1717 PMC4213622 25274364

[33] C. Blakemore , F. W. Campbell , J. Physiol. 1969, 203, 237. 5821879 10.1113/jphysiol.1969.sp008862 PMC1351526

[34] M. A. Georgeson , G. D. Sullivan , J. Physiol. 1975, 252, 627. 1206570 10.1113/jphysiol.1975.sp011162 PMC1348487

[35] S. Tagoh , L. M. Hamm , D. S. Schwarzkopf , S. C. Dakin , J. Vis. 2022, 22, 2. 10.1167/jov.22.11.2 PMC9547365 36194407

[36] C. Qian , Z. Chen , G. De Hollander , T. Knapen , Z. Zhang , S. He , P. Zhang , Hierarchical and fine‐scale mechanisms of binocular rivalry for conscious perception, *BioRxiv* 2023, 2023.02.11.528110, 10.1101/2023.02.11.528110

[37] P. Zhang , Y. Jiang , S. He , Psychol. Sci. 2012, 23, 254. 22301519 10.1177/0956797611424289

[38] F. Song , X. Dong , J. Zhao , J. Wang , X. Sang , X. He , M. Bao , ELife 2023, 12, RP93213. 10.7554/eLife.93213 PMC10937035 38478405

[39] D.‐z. Hu , X.‐y. Xie , C. Yu , P. Zhang , J. Vis. 2020, 20, 1749.

[40] J. B. Demb , G. M. Boynton , M. Best , D. J. Heeger , Vis. Res. 1998, 38, 1555. 9747491 10.1016/s0042-6989(98)00075-3

[41] J. B. Demb , G. M. Boynton , D. J. Heeger , J. Neurosci.: Offic. J. Soc. Neurosci. 1998, 18, 6939. 10.1523/JNEUROSCI.18-17-06939.1998 PMC6792964 9712663

[42] C. Müller‐Axt , A. Anwander , K. Von Kriegstein , Curr. Biol. 2017, 27, 3692. 29153326 10.1016/j.cub.2017.10.034

[43] C. Müller‐Axt , L. Kauffmann , C. Eichner , K. Von Kriegstein , Brain 2025, 148, 252. 39110638 10.1093/brain/awae235 PMC11706283

[44] N. Chaturvedi , E. T. Hedley‐Whyte , E. B. Dreyer , Am. J. Ophthalmol. 1993, 116, 182. 8352303 10.1016/s0002-9394(14)71283-8

[45] P. Zhang , W. Wen , X. Sun , S. He , Hum. Brain Mapp. 2016, 37, 558. 26526339 10.1002/hbm.23049 PMC6867378

[46] S. Gori , A. Facoetti , Vis. Res. 2014, 99, 78. 24325850 10.1016/j.visres.2013.11.011

[47] B. A. Sabel , J. Gudlin , JAMA Ophthalmol. 2014, 132, 381. 24504128 10.1001/jamaophthalmol.2013.7963

[48] B. Stojanoski , R. Cusack , J. Vis. 2014, 14, 6. 10.1167/14.12.6 25301014

[49] M. Kleiner , D. Brainard , D. Pelli , A. Ingling , R. Murray , C. Broussard , Perception 2007, 36, 1.

[50] M. Bach , Optom. Vis. Sci. 1996, 73, 49. 8867682 10.1097/00006324-199601000-00008

[51] D. L. Adams , J. C. Horton , Neuroscientist 2009, 15, 62. 19218231 10.1177/1073858408327806 PMC6913877

[52] Y. Qian , J. Zou , Z. Zhang , J. An , Z. Zuo , Y. Zhuo , D. J. J. Wang , P. Zhang , Proc. R. Soc. B: Biol. Sci. 2020, 287, 20200245. 10.1098/rspb.2020.0245 PMC7211434 32290803

[53] A. Schurger , J. Neurosci. Methods 2009, 177, 199. 18973774 10.1016/j.jneumeth.2008.09.028

[54] A. Delorme , S. Makeig , J. Neurosci. Methods 2004, 134, 9. 15102499 10.1016/j.jneumeth.2003.10.009

[55] J. R. Levin , R. C. Serlin , M. A. Seaman , Psychol. Bull. 1994, 115, 153.

