# Supplementary material for: Improving Adult Vision Through Pathway‐Specific Training in Augmented Reality
Source: Adv Sci (Weinh). 2025 Apr 7;12(21):2415877. doi: 10.1002/advs.202415877 (PMC12140354; doi:10.1002/advs.202415877)
Supplement: Supplementary file 1 — Supporting Information [file ADVS-12-2415877-s002.pdf]

ADVANCED SCIENCE
Open Access

Supporting Information

for *Adv. Sci.*, DOI 10.1002/advs.202415877

Improving Adult Vision Through Pathway-Specific Training in Augmented Reality

Yige Gao, Yulian Zhou, Qing He, Wen Wen and Peng Zhang**

Supplementary information

Table S1. Demographics and baseline characteristics.

	Age/ sex	Type of amblyopia	Amblyopic eye	BCVA at far (logMAR)		Current refractive errors	
				AE	FE	AE	FE
P01	29/F	Aniso	L	0.40	0	+1.00/-0.50*25	-6.25/-0.25*45
P02	25/M	Aniso	L	0.30	0	+5.75/-3.00*10	-1.50/-1.25*5
P03	26/F	Aniso	R	0.60	0	+1.75/-1.25*170	-3.75/-0.75*180
P04	34/F	Aniso	R	0.70	0	+2.50/-0.50*170	-3.25/-0.50*115
P05	23/M	Aniso	L	0.70	-0.08	+3.75/-0.75*30	0/-0.75*105
P06	21/F	Aniso	L	0.30	-0.18	+5.50/-1.75*25	0/-0.25*175
P07	25/F	Aniso	L	0.40	0	+2.50/-1.00*70	-2.25/-1.00*80
P08	30/F	Aniso	R	0.40	0	+2.50/-2.75*10	-4.25/-1.50*175
P09	30/F	Stra	R	0.30	0	-4.75/-1.50*170	-5.25/-1.00*165
P10	25/F	Aniso	L	0.52	-0.08	+4.50/-2.25*15	-1.00
P11	28/M	Aniso	L	0.30	-0.18	-9.25/-0.75*100	-4.00/-0.50*50
P12	30/F	Aniso	R	0.40	0	+2.00/-0.50*125	-2.75
P13	30/F	Stra	R	0.52	0	+2.75/-0.75*20	+3.25/-2.75*180
P14	26/M	Aniso	R	0.82	-0.08	+3.50/-0.50*50	-0.25/-0.50*180
P15	18/F	Aniso	R	0.22	-0.08	+2.50/-1.75*165	-1.75/-0.75*160
P16	29/M	Aniso	L	0.15	-0.08	+3.25/-0.50*180	-6.50
P17	26/F	Aniso	L	0.52	-0.08	+4.00/-2.00*45	0
P18	34/F	Aniso	L	0.30	0	+3.50/-0.75*60	+0.75
P19	25/F	Aniso	L	0.52	-0.18	+3.50/-2.00*20	-2.00
P20	37/F	Aniso	R	0.70	-0.18	+1.25/-0.50*130	-1.25
P21	29/F	Aniso	L	0.52	-0.18	+1.75/-0.25*55	-0.25
P22	21/M	Aniso	L	0.70	-0.08	+6.00/-1.00*15	-0.25/-0.75*180
P23	18/F	Mixed	L	0.70	-0.08	+8.00/-0.75*30	+0.75/-0.25*65
P24	23/M	Depr	R	0.70	-0.08	-0.25/-2.25*165	-0.50/-2.25*10
P25	21/F	Aniso	L	0.40	-0.08	+2.50/-1.25*40	-2.00/-0.75*180
P26	37/M	Aniso	R	0.60	-0.18	+6.50/-1.25*125	-3.75/-0.50*170

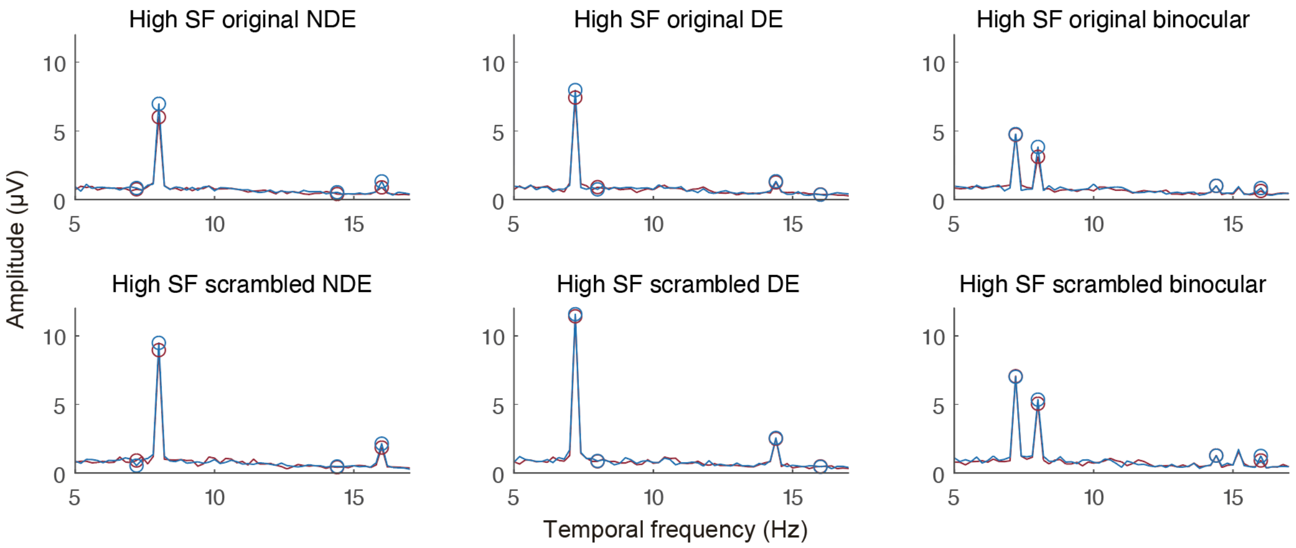

Figure S1. Group-averaged SSVEP amplitude spectra to high SF stimuli presented to NDE (8 Hz), DE (7.2 Hz) and both eyes. Red and blue lines show results in the pre-test and post-test sessions. Circles indicate SSVEP amplitude at the stimulus frequencies.

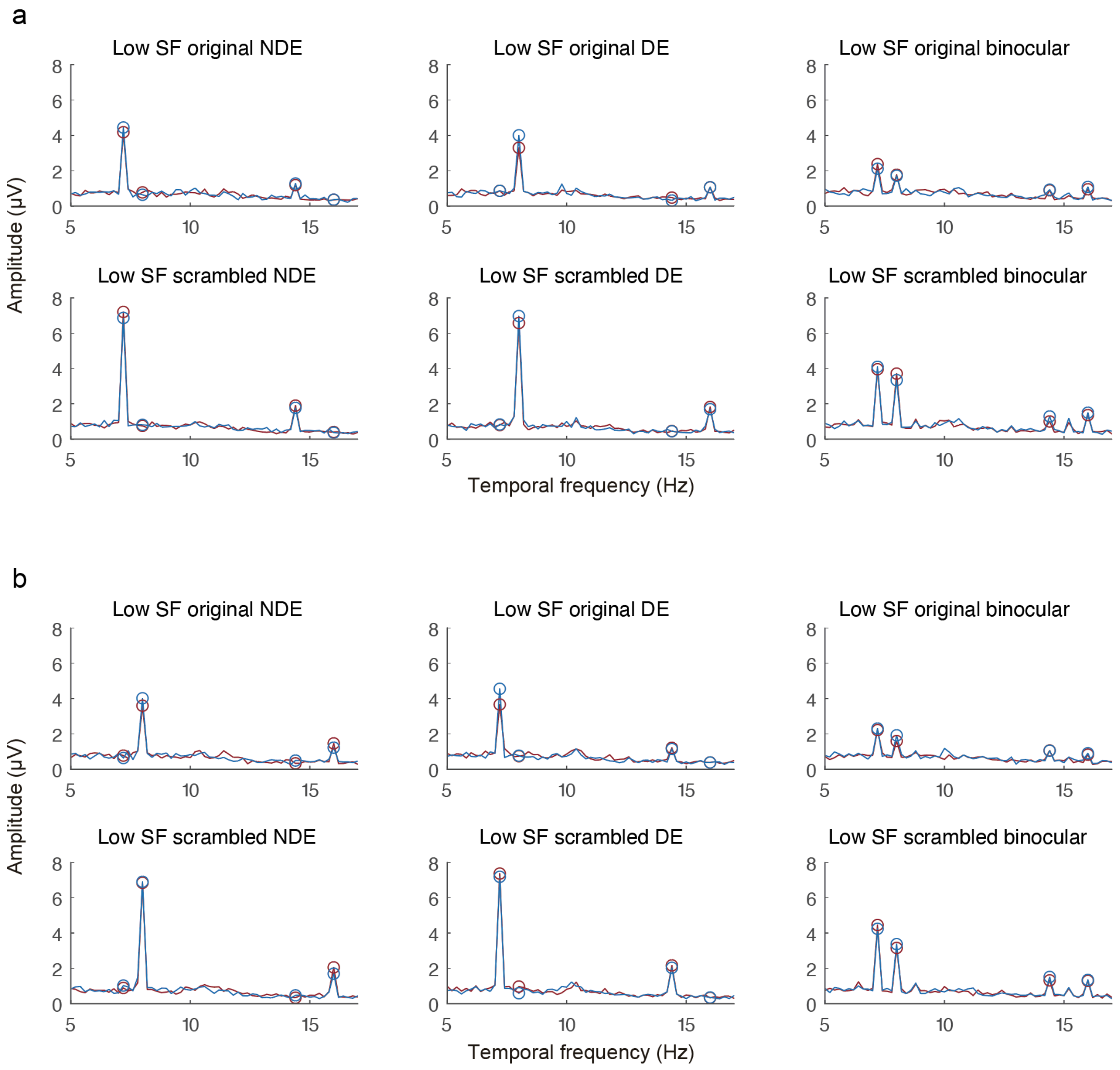

Figure S2. Group-averaged SSVEP amplitude spectra to low SF stimuli presented to NDE, DE and both eyes. NDE (DE) stimuli were presented at 7.2 (8) Hz in **(a)**, and 8 (7.2) Hz in **(b)**. Red and blue lines show results in the pre-test and post-test sessions. Circles indicate SSVEP amplitude at the stimulus frequencies.

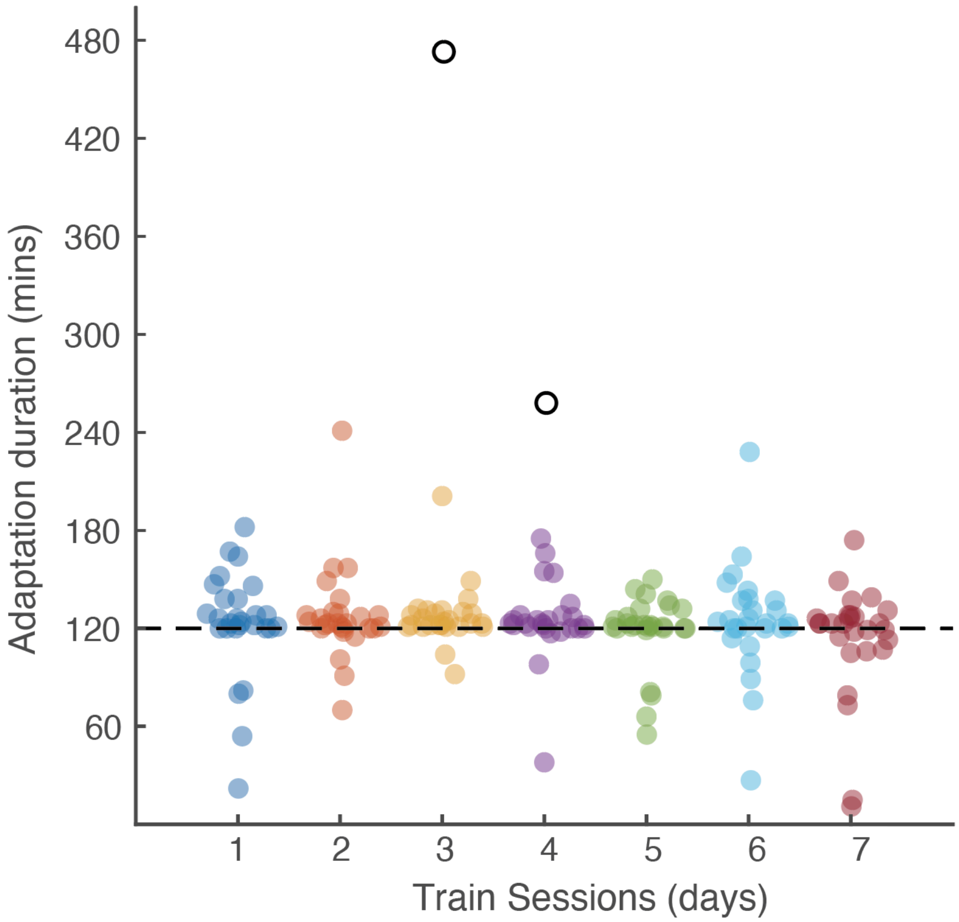

Figure S3. Adaptation duration in Exp. 3. Colored dots represent data from individual participants. Black circles indicate outliers from one participant who forgot to turn off the AR glasses after training. The dashed line indicates the prescribed adaptation duration (2 hours) in a single session.

Video S1. Fast flickering low-SF noise with intact high-SF stimuli presented to the weaker or amblyopic eye during adaptation. Low spatial frequency information was phase-scrambled as fast flickering noise (at 60 Hz in this example), while high spatial frequency information remained intact.

Video S2. High-SF image at low SNR presented to the stronger or fellow eye during adaptation. High spatial frequency information was phase-scrambled at 4 Hz and 100% amplitude, and then added to the high SF components with original phases and 50% amplitude.
